# *Himantoglossum adriaticum* H. Baumann × *Himantoglossum robertianum* (Loisel.) P. Delforge: A New Interspecific Hybrid Assessed by Barcoding Analysis

**DOI:** 10.3390/plants10010107

**Published:** 2021-01-06

**Authors:** Maurizio Antonetti, Stefania Nin, Gianluca Burchi, Stefano Biricolti, Massimo Gori

**Affiliations:** 1 CREA Research Centre for Vegetables and Ornamental Crops, Council for Agricultural Research and Economics, Via dei Fiori 8, 51012 Pescia, PT, Italy; stefania.nin@crea.gov.it (S.N.); gianluca.burchi@crea.gov.it (G.B.); 2 Dipartimento di Scienze e Tecnologie Agrarie, Alimentari Ambientali e Forestali (DAGRI), Università degli Studi di Firenze, Viale delle Idee 30, 50019 Sesto Fiorentino, FI, Italy; stefano.biricolti@unifi.it (S.B.); massimo.gori@unifi.it (M.G.)

**Keywords:** Orchidaceae, *Barlia*, breeding, in vitro germination, ITS, plastid markers

## Abstract

Most cultivated orchids, contributing to a worldwide highly profitable industry, are originated from tropic regions. Conversely, a considerable number of spontaneous orchids, belonging to the terrestrial orchids and widely diffused throughout the European continent, are not considered for trading due to their less gorgeous appearance and for technical difficulties in seed propagation. However, a breeding programme was undertaken aimed at developing a new hybrid between *Himantoglossum adriaticum* H. Baumann and *H. robertianum* (Loisel.) P. Delforge [syn. *Barlia robertiana* (Loisel.) Greuter] by applying techniques of anther conservation, manual pollination and in vitro asymbiotic germination of the obtained seeds. The plantlets that originated from the protocorms after seed germination were successfully acclimatised after potting in a proper medium. The parentage of the progenies of the hybridisation experiment was assessed by sequencing the Internal Transcribed Spacer assembly (ITS) and plastid barcoding markers of the parental lines and of the hybrids. The method proved to be effective in revealing the origin of the hybrids and to validate the maternal inheritance of the plastid DNA.

## 1. Introduction

Orchidaceae is the largest family of flowering plants comprising of approximately 32,000 species in around 800 genera. They are found in the majority of terrestrial ecosystems, from temperate grasslands to tropical rain forests [1,2,3] reflecting their extraordinary success in dispersing and adapting to a wide variety of habitats and environments. Two specialized groups have been recognised within the family: (i) the terrestrial orchids, which germinate underground in soil and represent around 25% of species, widely distributed in forest and grassland biomes; (ii) the epiphytes, which use other plants for mechanical support, constituting at least 70% of all known orchid species [4] and are found mainly in tropical forests.

Orchids account for a large share of global floriculture trade both as cut flowers and potted plants [5]. With their uniqueness of shape, breath-taking colours and exceptionally long shelf life provide a source of great aesthetic value. These qualities have made orchid production a highly profitable industry all-over the world. More than 100,000 hybrids are known globally and cultivated [6]. The ornamental orchid trade mostly consists of epiphytes originating from tropic regions, such as *Phalaenopsis*, *Dendrobium* and *Cymbidium* species. Among these, the cultivars of the genus *Phalaenopsis* have been among the world’s favourite flowering pot plants for many years [7]. Approximately a third of all wild orchids, however, are terrestrial [8]. All the spontaneous orchids found throughout the European continent, which are estimated at about 600 species [9], belong to this minor group. Some of these species, although generally having flowers of a smaller size compared to most popular cultivars, could gain a market space as ornamental plants for outdoor or cut flower, given the long pot life of their inflorescences. Nevertheless, the spread of these species on the market is hindered by technical difficulties in seed propagation. In fact, in the early stages of their life cycle, all terrestrial orchids are non-photosynthetic, totally lacking chlorophyll and relying on carbon acquired from a fungal symbiont for growth until the development of the first green leaves above ground, a nutritional strategy named mycoheterotrophy [10,11]. After initiation of the symbiosis, each embryo develops into a protocorm, a specialised organ that defines the first stage of germination. However, in in vitro culture some orchid species can be grown asymbiotically when sugars, amino acids and vitamins are exogenously provided in culture media [2,12,13]. The prodigious production of thousands of tiny seeds per plant containing minimal nutrient reserves for germination and growth leaves them totally dependent on a mycorrhizal fungal partner for the provision of carbon and mineral nutrients. The association with compatible endophytic fungi is species-specific, differing from species to species [14,15]. For instance, several mycorrhizal fungi, in particular *Basidiomycetes*, of the Tulasnellaceae family, and some species of Ascomyc etes, such as Helotiales, Sordariomycetes and Exophiala, have been found to be involved in the network with *Himantoglossum adriaticum* [16]. These symbiotic associations are somewhat problematic to recreate and maintain in co-cultivation during the in vitro propagation phases, hence, there is a need to develop protocols for asymbiotic in vitro seed culture on culture media provided with fundamental nutrients for germination. There has been a considerable increase in the number of studies on tissue culture over the past few years to support the goal of restoring endangered and threatened species and to promote commercial production and economic growth.

**Figure 1:**
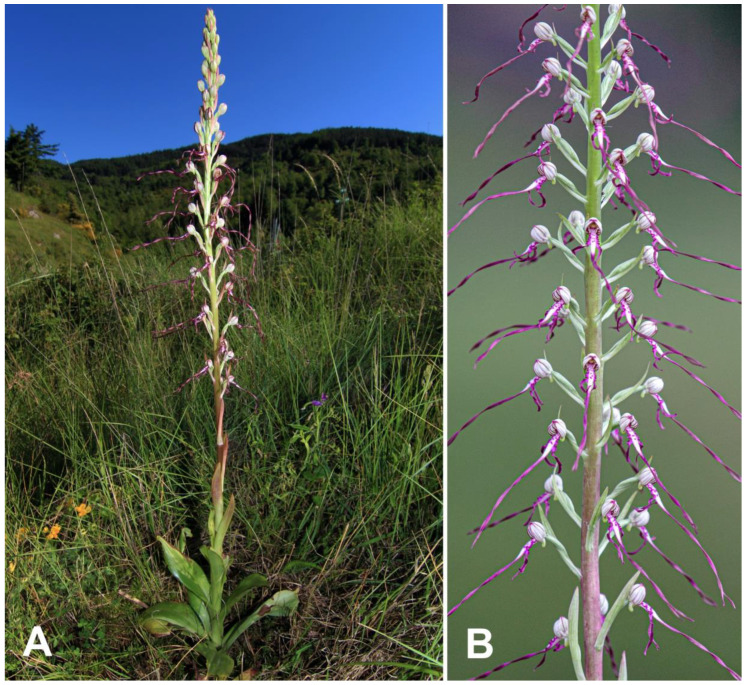
*Himantoglossum adriaticum*: whole plant in its natural habitat (**A**); detail of the inflorescence (**B**).

**Figure 2:**
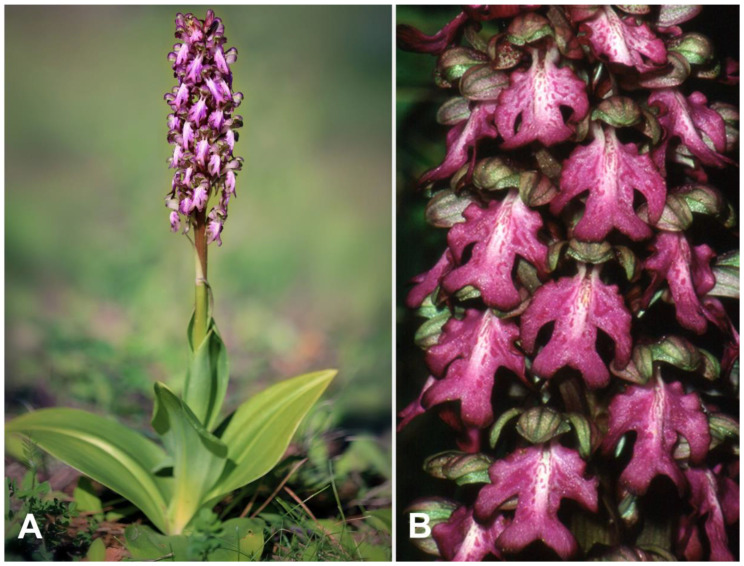
*H. robertianum*: whole plant in its natural habitat (**A**); detail of the inflorescence (**B**).

On these premises, as part of the RGV-FAO project (International Treaty on Plant Genetic Resources for Food and Agriculture), funded by MiPAAF (Italian Ministry of Agricultural, Food and Forestry Policies), a breeding programme was undertaken within the Eurasian genus *Himantoglossum* aimed at creating a hybrid, never before described between *H. adriaticum* H. Baumann (Figure 1) and *H. robertianum* (Loisel.) P. Delforge (=*Barlia robertiana*) (Figure 2), by anther conservation and manual pollination, and at confirming the hybrid nature of the obtained protocorms by molecular analysis of the Internal Transcribed Spacer (ITS) region of nuclear ribosomal DNA and plastidial DNA barcoding sequences (as detailed in Section 2.5 Molecular Analysis).

The inflorescences of both parent species are some of the most striking in Europe thanks to their size, uniqueness of shape and colours. Both species are vegetatively robust and produce long racemes of large flowers that are characterized by distinctive, unusually elaborate labella. *H. robertianum* can reach heights up to 50–80 cm and this feature has earned it the common name ‘Giant Orchid’ in the Anglo-Saxon countries; *H. adriaticum*, known as ‘Lizard orchid’, has an even more slender bearing, than in some specimens can exceed 1 m in height. 

The charismatic *Himantoglossum* s.l. clade of Eurasian orchids contains an unusually large proportion of taxa that are of controversial circumscriptions and growing conservation concern [17]. *H. robertianum*, for example, belongs to the subgenus *Barlia*, previously considered a separate monotypic genus. The inclusion of *Barlia* within *Himantoglossum* was advocated by Delforge [18] and then supported molecularly by Bateman et al. [19]. However, many authors of European orchid floras and monographs, including the most recent checklist of Italian flora [20], still prefer the ‘traditional’ classification [17] and consider *Barlia* as a distinct valid genus. In this latter case, the new hybrid described above should be considered as intergeneric. 

Orchid species are particularly prone to hybridization because of the high number of sympatric species and a general lack of complete reproductive barriers [21,22]. Therefore, together with several processes (vicariance, polyploidization, etc.), hybridization represents one of the most important drivers for speciation within the Orchidaceae family, in particular in Mediterranean species [23]. Moreover, floral isolation is a form of prepollination reproductive isolation that can play an important role during the process of plant speciation, and, in the absence of geographic barriers to gene flow, can be the most important prezygotic barrier [24]. The increasing development and availability of suitable molecular markers have facilitated studies of natural hybridization, allowing to detect even low levels of introgression and to provide information about the potential gene flow and reproductive barriers between species [25].

In addition to the existence of the artificial hybrid *H. jankae* × *H. robertianum* and the natural hybrid *H. ×samariense* (*H. comperianum* × *caprinum*), traces of natural hybridization show the possibility of gene flow between *H. adriaticum* and *H. hircinum*; *H. adriaticum* and *H. jankae*; and within the *H. jankae-caprinum* clade [17]. However, so far, there is no evidence of the existence of gene flow between *H. adriaticum* and *H. robertianum*, also due to an evident asynchrony in their flowering period.

Interest in the *Himantoglossum* s.l. clade is by no means confined to taxonomic issues. Along with many other European orchid species, *Himantoglossum* taxa have been studied at least superficially for their pollinator spectra [26] and for their long-term, quantitative population demographics [27,28,29] and phenology [30,31].

*H. robertianum* belongs to the steno-Mediterranean chorotype, with a large distribution area, spanning from Portugal and Morocco to Anatolia. In the last decade, climate changes have probably favoured the gradual shift of this species towards higher latitudes [32]. It is the earliest Italian orchid to bloom. In the coastal areas of the southern part of its distribution area, anthesis has been reported to occur in early November, but generally, the peak bloom takes place in winter from January to April, when demand for flowers is higher. Its variability is limited to the different shades of flower colours. Although the species has been found to expand its presence across the Mediterranean distributional range, it is included in the Appendix II of the Convention on International Trade in Endangered Species of Wild Fauna and Flora (CITES), and as Least Concern on the European red list [33]. In Italy it is a protected entity at national level and is covered by total regional protection in different areas. In fact, since this plant is favoured for its conspicuous appearance, remarkable vase life of cut flowers and pleasant scent, many people pick it from its natural habit for personal use [34].

*H. adriaticum* is an Adriato-Mediterranean endemic species with a distribution in Eastern Austria, Bosnia-Herzegovina, Croatia, Czech Republic, Hungary, Italy, Slovakia, Slovenia [16,35,36]. In Italy it is found mainly in the central regions, while is rare in the north and south part of the country and completely absent in the two major islands. Unlike *H. robertianum*, it blooms between end of Spring and early Summer (May–July) [32,37]. It is included, as Least Concern (LC), on the International Union for Conservation of Nature (IUCN) [38] and on the Italian [39,40] Red List of threatened species.

Thanks to the intrinsic value of plant novelties, interspecific hybridization represents a useful tool in ornamental breeding. Although the expression of morphological traits in hybrids is unpredictable, it often results in a mosaic of parental, intermediate and new characters [25,41]. Considering the above-described peculiarities of both parental species, the ultimate goal of our breeding plan was to introduce valuable traits for new potted and garden flowering plants, or cut flowers. Of course, the quality of the obtained seedlings could be evaluated with appropriate tests only when the anthesis stage will be reached, likely in a couple of years.

Moreover, availability of commercial varieties might prevent the flower enthusiasts from picking the wild species [42]. Indiscriminate collection and habitat destruction are constantly increasing and represent one of the major extinction threats of European orchids. This is particularly true for showy species of conservation concern, as is the case with species belonging to both subgenera *Barlia* and *Himantoglossum.*

Here we report the successful interspecific crossing experiment in *Himantoglossum* using DNA sequence diversity of the internal transcribed spacer (ITS) region of nuclear ribosomal DNA and plastid barcoding marker *rbcl*, *matK* and *psbA-trnH* intergenic spacer from *H. adriaticum* and *H. robertianum*, and their putative hybrids for genetic analysis.

## 2. Results

### 2.1. Cross-Pollination and Capsule Development

**Figure 3:**
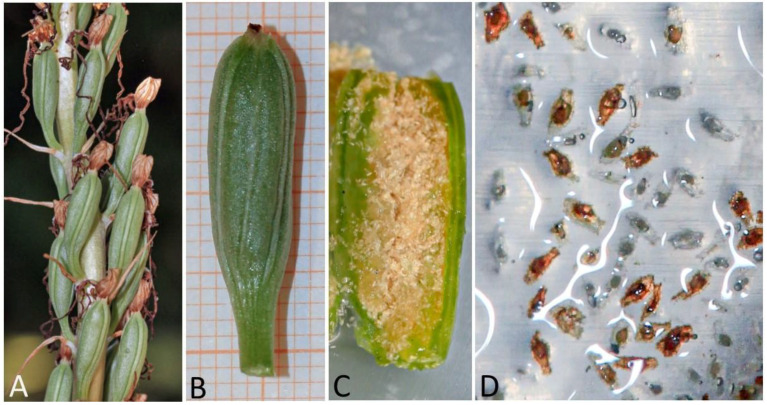
*H. adriaticum*: immature infruttescence (**A**); capsule at the optimal maturation stage for culture establishment, corresponding to a length > 20 mm and a thickness > 5 mm, having the external surface still green and the partitions intact (**B**); cross-section of the same orchid capsule showing immature seeds inside (**C**); seeds at different ripening stage taken from the capsule (**D**).

All *H. adriaticum* flowers hand cross-pollinated with *H. robertianum* pollinaria were successfully fertilized, showing a rapid development and an evident swelling of the capsules (Figure 3A). Self-fertilization of the mother plant was prevented by emasculation of the entire inflorescence before anther dehiscence and pollen maturity. Being *H. adriaticum* a strictly entomophilous species, the inflorescence was then covered with an anti-insect tulle bag to avoid the female being pollinated by stray pollen carried by insects. 

### 2.2. In Vitro Sowing and Germination

**Figure 4:**
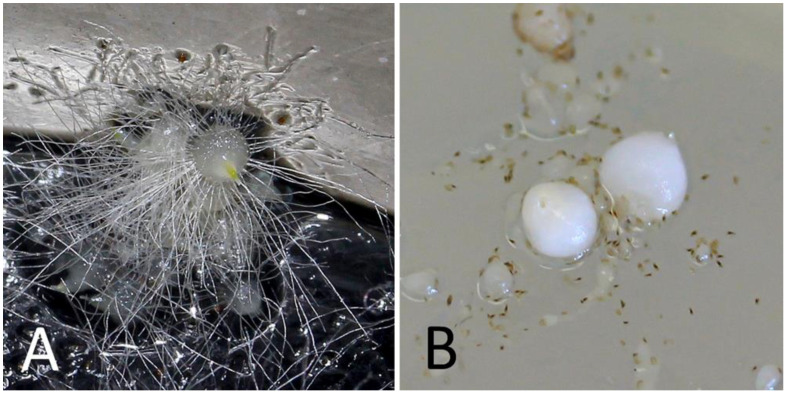
Globular protocorm at stage 4, with a diameter greater than 1 mm and well-developed rhizoids onto M551 medium (**A**); detail of stage 4 protocorm obtained onto the BM-1 medium without basal rhizoid formation (**B**).

More than 9500 protocorms were obtained from in vitro seed germination processes, with an average of about 600 shoots per capsule (Table 1). Results showed that both tested media supported the germination of *H. adriaticum* × *H. robertianum* seeds, although M551 seemed to be more effective compared to BM-1 medium for hybrid germination and growth, giving rise to about 60% protocorm development to the stage of ‘rhizoids’ production (Figure 4). Fungal contamination occurred in 3 pairs of jars out of 64 jars (C6 and C62 onto M551, C59 onto BM-1) as shown in Table 1. Capsules harvested 28 days after pollination resulted in the largest amounts of viable seeds/developing protocorms (1217 vs. 187–380), although capsules collected at different times (21, 23, 26, 27 and 28 days) from hand cross-pollination did not present notable changes in colour or pubescence degree, nor visible differences were noted in the seed appearance and consistency at the time of capsule opening.

On average, protocorms reached the stage of swollen embryo (diameter of ≥1 mm) with simple rhizoids after 18 weeks of in vitro culture, but development seemed to be more advanced on M551 than BM-1 medium, with a range of 12–22 weeks and 13–23 weeks, respectively (Table 1). When stage “rhizoids” were obtained on the BM-1 medium, basal rhizoid formation was less evident or even suppressed in the most extreme cases (Figure 4).

### 2.3. In Vitro Establishment and Cultivation

Few weeks after transferring on M551 medium, all the protocorms became quickly similar in appearance, regardless of the type of substrate used for seed germination process. No vegetative multiplication was observed. Time needed for individual protocorm development proved to be extremely variable even among seedlings originating from the same capsule, probably due to the different initial degree of embryo maturation at the time of in vitro seeding. After the tenth subculture, some seedlings showed symptoms of leaf browning and necrosis of root tissues.

### 2.4. Acclimatization

After 9 weeks under standard ambient condition (room temperature and natural daylight) and plastic film protection, the rate of *H. adriaticum* × *H. robertianum* seedling survival was 100% and the aerial part of all plantlets remained vital until the end of the vegetative phase, which in our experimental condition took place in June 2019. The effective assessment of plant acclimatization was recorded at the restart of the growing season. Nineteen % of the plants were successfully acclimatized over a period of approximately three months, from 28 October to 7 February 2019.

### 2.5. Molecular Analysis

The complete Internal Transcribed Spacer assembly (ITS1-5.8S-ITS2) of the nuclear ribosomal DNA and the plastid barcoding DNA sequences (*rbcl*, *matK* and *psbA-trnH* intergenic spacer) were amplified with the DNA extracted from the samples as template. A single band was detected in the agarose gel in all samples, as expected (Figure S1). The amplicons have been directly and bi-directionally sequenced by using the corresponding primers for each amplified sequence. 

The samples of *H. adriaticum* and *H. robertianum* produced single sequences, also for the nuclear ITS DNA, confirming the amplified locus in both parental species was homozygous. Indeed, direct sequencing of the amplicon obtained from the two germinated seeds originated from the fertilisation of *H. adriaticum* with *H. robertianum* pollen, showed the presence of two overlapping sequences [43]. Sequencing output coincided only for the initial part of the sequence, indicating a shift of the reading frame due to the presence of insertions. The presence of different sequences within the ITS amplicon led to suppose a putative hybrid origin of the samples. To assess the parental species originating the putative hybrid, the ITS PCR fragment was cloned to isolate the sequences composing the amplicon. The sequences of eight recombinant colonies obtained from cloning the fragment of the two putative hybrid seedlings were aligned with those of the putative parents. The colonies produced sequences corresponding either to the one of *H. adriaticum* or to that of *H. robertianum* as reported in Table 2 confirming the hybrid origin of the seedlings. As far as the plastid DNA sequences are concerned, the *rbcl* fragments of the hybrids showed a complete homology with *H. adriaticum*, while two single nucleotide polymorphisms were detected in *H. robertianum* supporting the hybrids’ maternal inheritance of the plastid DNA. In addition, the sequences of *matK* in the hybrids showed some point mutations in comparison with *H. robertianum* and a complete overlapping with *H. adriaticum*. The same sequence overlapping with *H. adriaticum* has been observed with *psbA-trnH* intergenic spacer of the hybrids, while significant triplet deletions and insertions occurred in the comparison with *H. robertianum.* Table 3 resumes the comparative information for plastid DNA surveyed barcodes.

These observations confirm the different discrimination capability of the three plastid barcoding sequences [44] and strongly suggest the maternal inheritance of the hybrids’ organelles.

## 3. Discussion

The purpose of the present work was to develop a new interspecific hybrid between species of different subgenera (*Barlia* and *Himantoglossum*), which are morphologically and genetically distant [17] and even today are considered by some authors as separate genera due to their large morphological differences [20,34].

To date, very few natural hybrids have been described between species belonging to different subgenera (or ex genera) of *Himantoglossum*, including *Himantoglossum* × *agiasense* Karatzas (ined.), hybrid between *H*. *comperianum* and *H. montis-tauri* (previously described as *× Comptoglossum agiasense* Karatzas). To our knowledge, no research papers for artificially created hybrids between the subgenera *Barlia* and *Himantoglossum* have been reported in worldwide literature until now. The hybrid obtained in this study cannot be generated even in nature, due to the action of non-specific pollinators, as the anthesis period of the two species differs by several months. Furthermore, the distribution area of the two species do only partially overlap, as continental Italy is the only region of Europe in which the two species coexist [45,46], though rarely in the same areas, since their soil and climate requirements are quite different [34,37].

Flower visiting pollinator communities observed in the field are distinct for each species. While *H. robertianum* is visited by multiple pollinators, including *Xylocopa violacea*, *Bombus* spp. (Family Apidae) and *Osmia cornuta* (Family Megachilidae) [47], only few species of the genus *Colletes* sp. (Family Colletidae) have been found to be active flower visitors in *H. adriaticum*. This makes it even more difficult that the two species interbreed naturally.

The use of artificial hybridization techniques in plant breeding remains one of the most powerful tools for obtaining new characters in ornamental species and transferring desirable agronomic characteristics to commercial varieties [48]. In this specific case, the full genetic compatibility between the two species (*H. adriaticum* and *H. robertianum*) is demonstrated by both the abundance of obtained seeds and their relative ease of germination. Hence, our results represent a further element in favour of the recent inclusion, not yet universally accepted, of the former *Barlia* genus within the *Himantoglossum* genus. It will be interesting to observe whether the hybrid will be sterile or fertile once it has reached the anthesis stage. Since orchids from the Mediterranean region regularly form natural interspecific (and sometimes intergeneric) hybrids that are highly fertile, it is very likely that the developed *H. adriaticum* × *H. robertianum* hybrid will be fertile. 

The applied anther conservation technique proved to be effective in guaranteeing the viability and germination capacity of the pollen grains of *H. robertianum* for a period of over two months. Ovule fertilization occurred in up 100% of hand-emasculated flowers artificially pollinated with whole preserved anthers, developing seed capsules of normal appearance, containing thousands of seeds (Figure 3).

Molecular analysis of the ITS1-5.8S-ITS2 regions of the nuclear ribosomal DNA of the two different putative hybrid seedlings clearly demonstrated the hybrid origin of the protocorms and that *H. adriaticum* and *H. robertianum* are the parental species, excluding the possibility that the seeds were obtained by apomixis processes. The plastid barcoding DNA sequences (*rbcl*, *matK* and above all, *psbA-trnH* intergenic spacer), suggested a *H. adriaticum* maternal inheritance of the two hybrids’ plastids. In addition to their use in assessing structure and diversity in molecular phylogeny, the analysis of the ITS region has proved to be a reliable tool for indicating the parentage of hybrids, provided that evidence of the hybridisation is available [49]. Furthermore, as evidenced by Grassi et al. [50], plastid DNA analysis provides a convenient and efficient tool to validate the maternal origin through the analysis of uniparentally inherited genes. 

The present study was not specifically focused on seed quality assessment, but rather it was aimed to obtain as many hybrid protocorms as possible under asymbiotic germination conditions. Thus, any potentially destructive intervention that could either reduce seed availability or somehow compromise the sterility conditions of the in vitro seeding process, was limited to the minimum necessary. For the same reason, seed viability test was not performed, and the seed germination rate was not calculated. Previous reports on seed quality assessment indicated that the efficacy of vital stains using Triphenyl Tetrazolium Chloride (TTC) to determine *H. adriaticum* seed viability, has to be considered uncertain, as significant disagreement between degree of staining and both in situ and ex situ (in vitro) germinability was observed [51]. Both TTC and Fluorescein Diacetate Assay (FDA) have often produced variable results in assessing seed viability in some other orchid species [52,53,54] and the correlation between observed germination and TTC/FDA staining was shown to be species specific for terrestrial orchids, with FDA generally providing a more accurate estimate of viability than TTC. Optimal seed pre-treatments should be determined for each species before testing viability by these methods, and viability estimates should always be corroborated with germination studies when working with temperate terrestrial orchids [54,55,56].

Given that the inside of immature capsules is considered sterile, the procedure for effective sterilization requires only the outer surface disinfection of the capsules. It should be mentioned here that when naturally sterile seeds from immature capsules are used for asymbiotic seed germination, it is difficult to properly assess the number of seeds out of the total of those being transferred on germination media that are naturally capable of germinating. This is related to an existing ripening gradient inside the capsule. Indeed, in vitro germination cannot take place both for overripe seeds, which have already developed a relative impermeable seed coat preventing the entry of water and nutrients, and for immature seeds, whose embryo development fails to occur on artificial media. Differences in seed maturity stage are partially visible in Figure 3D. Consequently, the initial amount of germinable seeds largely depends on the degree of capsule maturation. This extent of putative developing embryos cannot be quantified and even deducted from the days between flower pollination and capsule collection, as this period is influenced by imponderable factors such as climatic conditions and the number of pollen grains really penetrated into the ovary. Moreover, the need to maintain sterility cultural conditions, as well as the extremely small seed size, further hampered the assessment of seed amounts placed on the tested germination media.

Seed morphometric studies on the parental species have shown that *H. adriaticum* and *H. robertianum* have quite similar seed size, 531 ± 54 µm × 135 ± 25 µm for the seed-bearing species [57] and 350–530 × 150–210 µm for the pollinating species [35], respectively. Each capsule can contain a very large and extremely variable quantity of seeds; according to Bódis et al [35], the estimated number of seeds per capsule of *H. adriaticum* can vary between 1119 and 23,740. Taking into account that 1473 protocorms have been obtained starting from a single immature capsule (C26, Table 1), which obviously contained a number of zygotic embryos that was unsuitable for asymbiotic in vitro germination (under- or over-developed), it is realistic to assume that the adopted protocol is very efficient for obtaining a large production of seedlings. This is even more true when considering that a potential decrease in fertility could have been expected by genetic incompatibilities between two so phylogenetically distant species. In addition, seeds did not require any vernalization treatment (4 °C for 3 months), as described in the literature for in vitro germination of *H. adriaticum* fully ripe seeds [51]. Although both tested media supported seed explant germination and development, M551 medium was the most effective for seed germination and protocorm growth of *H. adriaticum* × *H. robertianum*, giving rise to globular protocorm of more than 1 mm of diameter with absorbing hair formation within 18 weeks of culture initiation, on average. Similar findings were also reported in previous studies on seeds germination of other orchid species found in Italy. Seeds of *H. adriaticum* needed 8–11 months after sowing to germinate in their natural habitats, whereas during ex situ germination, the first protocorm appeared 7 and 9 months after sowing on modified Fast media at pH 5.5 and pH 7.5, respectively [35]. Seeds collected from immature capsules of twenty-eight taxa of Italian wild orchids successfully germinated under asymbiotic conditions and reached the stage of protocorm with leafy shoot in a period variable between 11 and 45 weeks after sowing [58]. Compared to the aforementioned literature reports, the time interval needed for hybrid seeds to develop into protocorms can be regarded as satisfactory, also in view of possible future small-scale commercial production of hybrid plants.

No multiplication of the plant material was observed during in vitro cultivation, not even by dissecting the rhizotubers. The protocorms reached the optimal size for transfer of plantlets from in vitro to ex vitro conditions by increasing both in the aerial part (formation of leaflets) and in the hypogean part (formation and enlargement of the rhizotubers), with emission and lengthening of new rhizoids, without however increasing in number.

Achieving a balance between biodiversity conservation and market demand has become an urgent task for orchid conservationists world-wide. Consideration of symbiosis is one of the key factors for the restoration of declining orchid populations. Typically, the orchid industry uses asymbiotic tissue culture to meet large-scale market demand for ornamental products where an abundance of seedlings can be generated over short periods of time and under controlled conditions. Large scale ex situ production, however, often requires techniques that are labour intensive to acclimatize and transplant propagules. Moreover, the seedling obtained via asymbiotic germination often result in low survival rates in the field. For the acclimatization of the asymbiotically propagated seedlings, the use of soil taken from orchid natural habitat might be useful to ensure optimal conditions (pH, nutrients, etc.) that favour both the development of symbiotic fungi and the growth of orchid propagules and might result in better adaptation to the environment leading to higher survivorship and faster growth. This could facilitate the reintroduction of selected endangered species to wild habitats and overcome the fact that seedling sources are often a bottleneck in the process of commercial orchid cultivation. However, the use of non-renewable natural resources for economic growth [59] is unsustainable, since it involves a disturbance of endangered plant habitat and does not allow a standardization of cultivation methods. Alternative substrates, such as the formulation used in the present work, have been efficient for the cultivation of seedlings of orchids. Previous research findings have evidenced that some terrestrial orchid species can be successfully established in alternative standardized substrates, since they are able to take advantage from the presence of non-specific saprophytic fungi present in the substrate soil [60]. In particular, the formulation described in the present work has shown great potential for the acclimatization and therefore has been chosen as ex vitro propagation substrate in the present study. 

In order to clearly assess hybrid establishment after transplanting under ex vitro condition in the greenhouse, we had to wait for growth cycle starting again. In fact, in the absence of specific biotic and abiotic stresses, orchids that receive enough water typically keep the leaves turgid during the first months of acclimatization in the shade, even if plants have not yet developed a proper root system. The dry season represents the most critical phase, and this is the time when orchid species stop vegetative growth. Shoot initiation occurs in the early rainy season. In our study, only the shoots overcoming the dormant (resting) phase, entering in the new growth and developing new leaves in the following autumn, were considered successfully acclimatized to ex vitro conditions. As a first result of the research on hybrid seedling growth without the presence of natural symbiont fungi, a survival rate of almost 20% may be considered satisfactory in order to obtain a sufficient number of seedlings for agronomic and phenotypic assessments of the new hybrid.

An important aspect of this new hybrid is the great ornamental potential of its flowering period. In fact, one of the two parental species, *H. robertianum*, reaches full anthesis within its range in late Autumn to Winter, when the array of potted and garden plants blooming exuberant and fragrant flowers available on the market is very limited. If hybrid earliness should be confirmed in the following years, this trait could represent an important added value for ornamental production. What will be the flowering period of the hybrid is nearly impossible to predict at the moment.

In addition to its ornamental potential discussed so far, the newly created hybrid may represent an important source of nutrients in the food industry, as for the production of flour, cream, confectionery and beverages of great nutritional and cultural relevance [61,62]. In some regions bordering the Eastern Mediterranean, from Greece to Albania, Iran to Turkey, species belonging to both the subgenres *Barlia* and *Himantoglossum* [63] are traditionally used for the preparation of a flour called ‘salep’ (or salehp or salepi) particularly rich in glucomannans, because are those with the largest rhizotubers among the European orchids. The production and consumption of salep have strongly increased in recent years, also due to exports to Germany and other Western European countries, causing a perturbing intensification in the number of wild orchid pickers and illegal excavation of tuber [64]. Orchid tuber collection in Turkey alone has been estimated to annually use fresh tubers from 30 to 120 million orchid plants producing over 115 tonnes of salep, whereas in Iran between 5.5 and 6.1 million orchids are destroyed every year [63,64].

Taking into account that hybrid vigour, or heterosis, is generally higher than that of the parent species, it is realistic to expect that the new hybrid described in this work might be a yield potential of semi-finished products of standardized quality for the food industry. Thus, it will contribute to the protection of threatened orchid habitats with a high risk of extinction.

## 4. Materials and Methods

### 4.1. Interspecific Hybridization

#### 4.1.1. Anther Conservation

In situ natural growing *H. robertianum* was used as pollen donor. A patch with an approximate surface area of 10 m^2^ was defined in an area located in the province of Livorno in central Italy (Tuscany). This area was selected as a sampling site as it hosts one of the biggest *H. robertianum* populations. Fifty pairs of mature pollinaria, comprising pollinia and caudicles (Figure S2), were manually collected from fresh inflorescences at the full bloom stage on 16 March 2017. The anthers were dehydrated with silica gel and stored for 70 days in the dark, at 4 °C, inside sterile Petri dishes sealed with parafilm. Twenty-four hours before pollination, the pollen was rehydrated by placing them on discs of filter paper moistened with sterile deionized water [65].

#### 4.1.2. Cross-Pollination

A spontaneously growing plant of *H. adriaticum* was used as mother plant. The plant was identified in a plot of uncultivated land within a private property in an area of Chianti hill countryside located in the province of Florence. The plant was labelled and protected by a metal mesh, to avoid any damages by wild animals (wild boars, porcupines, etc.). Shortly before anthesis, the inflorescence was hooded with very light white tulle, preventing pollinating insects (apoid hymenoptera insects that belong to the Colletidae [47] family) to have access to the flowers. Each opening flower of the inflorescence was manually emasculated in order to avoid any possibility of self-fertilization. The emasculated flowers were then manually pollinated by rubbing an entire pollinarium of *H. robertianum* inside the stigmatic cavity so as to ensure the deposition of the pollen grains, leaving the pollinarium adhered to the base of the gymnostemium (Figure S3). The pollinated flowers were numbered, from the bottom upwards, following the natural order of inflorescence anthesis. Totally, 65 flowers were emasculated and pollinated in one day (30 June 2017).

#### 4.1.3. In Vitro Sowing and Germination

N.16 capsules produced by the fertilized flowers (capsules n. 1–7 and 9 in the basal part, capsules n. 10–13, 15, 20 and 26 in the median part, capsules n. 59 and 62 in the apical part) were collected over a period of 21–28 days after pollination. The optimal maturation stage for culture establishment corresponds to a length > 20 mm and a thickness > 5 mm, having the external surface still green and the partitions intact, as shown by previously in vitro seeding tests [58] (Figure 3). The day after harvest, capsules surface disinfection was carried out in a laminar flow hood as follows: dipped in a mixed solution (*v*/*v*) of sodium hypochlorite (5%) and Tween 20 (0.2%) for 30 min and then rinsed three consecutive times with sterile distilled water. The culture media used for in vitro germination were M551 (Malmgren modified terrestrial orchid medium without sucrose) and BM-1 (Terrestrial orchid medium with vitamins, sucrose and casein hydrolysate) produced by PhytoTechnology Laboratories^®^, Shawnee Mission, United States [66]. The medium was adjusted to pH 5.9, solidified with 5.5 g/L agar (Plant Agar purchased by Duchefa Biochemie, Haarlem, The Netherlands) before autoclaving for 20 min at 121 °C and 108 kPa. The content of each capsule was accurately removed and placed in four 170-mL glass jars, two of each containing either M551 medium or BM-1 medium, supplemented with 0.1 mg L^−1^ BAP, with ovaries randomly and as equally as possible represented within and across the two substrate types. Cultures were incubated in growth cabinet with a precision thermoregulator (±1 °C) at temperature regimes of 21 °C in continuous darkness. Darkness was obtained by placing jars in closed cardboard boxes. Germination was assessed at weekly intervals with an overall duration of 4 months. The criterion for germination was the achievement of the globular protocorm stage with a diameter greater than 1 mm, with well-developed rhizoids (Figure 4). This stage corresponds to stage n^o^ 4 (‘rhizoids’) of embryonic development [67,68,69].

#### 4.1.4. In Vitro Establishment and Cultivation

**Figure 5:**
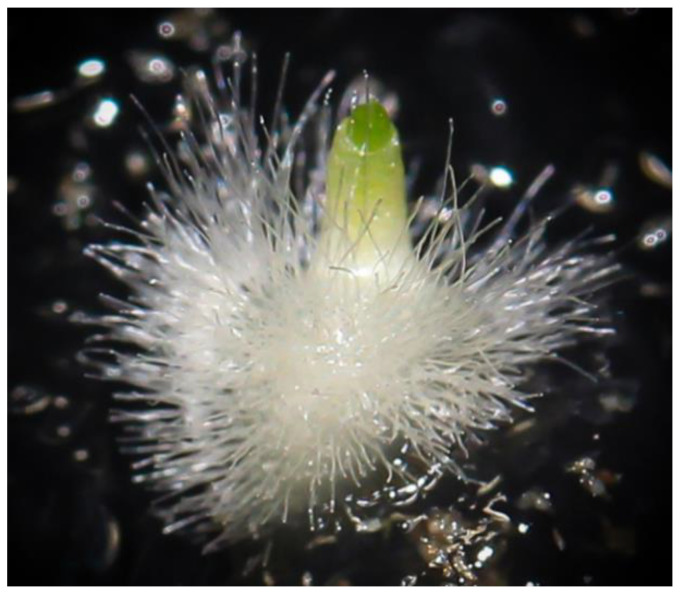
Protocorm at the stage of shoot initiation.

When the protocorm stage of shoot initiation was achieved (Figure 5), cultures were maintained at 21.0 ± 1.0 °C under a 16/8 h photoperiod with a photon flux density of 35 µmol m^−2^ s^−1^ at the culture level provided by pairs of cool-white fluorescent lamps. After two weeks, the protocorms were transferred onto fresh M551 medium containing 0.1 mg L^−1^ BAP. Subsequently, explants were subcultured onto fresh medium of the same composition every two months to maintain active growth and select healthy cultures.

#### 4.1.5. Acclimatization

**Figure 6:**
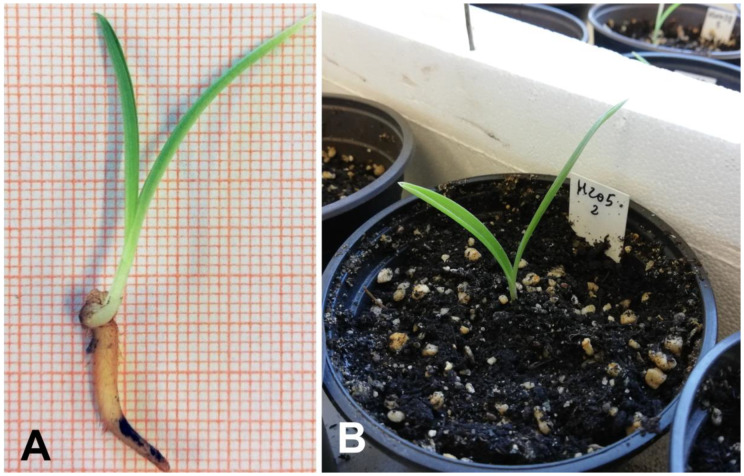
Stage of two leaflets development: well-formed plantlets with one of the two leaflets, sized at least 3 cm, selected for the acclimatization test (**A**); plantlets in pots containing commercial peat-based acid substrate, sand and perlite (70:20:10 *v*/*v*) (**B**).

Once the stage of two leaflets development was reached, 100 well-formed protocorms having one of the two leaflets of a size of at least 3 cm (Figure 6A) were selected for the acclimatization test. Based on different maturity levels of seeds within the capsules and scalar seed germination and protocorm development, the experiment was carried out in two successive stages, on mid-January and mid-February 2019. Protocorms were planted in pots of 10 cm diameter, containing commercial peat-based acid substrate, sand and perlite (70:20:10 *v*/*v*), modified from the formulation described in [59] (Figure 6B). This substrate formulation was previously used successfully for the acclimatization of other spontaneous orchid species belonging to the genus *Anacamptis* Rich. and *Serapias* L. [58]. Pots were first placed for 9 weeks inside expanded polystyrene containers and stored under a transparent polyethylene film at room temperature (10–24 °C), and natural daylight. Temperature and humidity were monitored by a digital thermo-hygrometer. Thereafter, all plants were acclimatized by lowering the relative humidity from initial values, close to saturation (<99%) to values below 30% by gradually removing the protection over 2 weeks. Subsequently, the seedlings were transferred outdoors, under a shade house (90%), where they were kept until the end of the vegetative phase (June) and the subsequent recovery, in Autumn. During the Summer season, the substrate was kept slightly humid with 60 seconds wetting every week. Survival percentage was recorded at the vegetative restart in Autumn.

### 4.2. Molecular Analysis

#### 4.2.1. Sample Collection and DNA Extraction

The genotype of the seedling originated from cross-pollination of *H. adriaticum* × *H. robertianum* was determined by DNA barcoding analysis on either the parental species either the putative hybrid seedlings. Newly formed young leaves (totally four samples) were taken from the same *H. adriaticum* mother plant and *H. robertianum* population selected for crossing experiment as seed-bearing and pollen donor plants. The sampled leaves were stored in falcon tubes containing 70% ethanol in order to preserve DNA integrity of plant material during transportation to the laboratory, where samples were kept in the fridge until submitted to DNA extraction. Leaf samples collected from two seedling lines derived from the in vitro cultivation test were stored in the freezer (−20 °C). The samples stored in ethanol were previously dried under vacuum overnight and the total genomic DNA was extracted from approximately 50 mg of leaf material using the DNeasy Plant Mini Kit (Qiagen, Hilden, Germany), according to manufacturer’s handbook. The quantification and purity of extracted DNA was carried on by measuring the absorption at 260 nm and 280 nm using a TECAN spectrophotometer (TECAN group, Männedorf, Switzerland). In order to verify the integrity of the DNA, 3 µL of extracted DNA were run on a 1.0% (*w*/*v*) agarose gel (Sigma Aldrich, St. Louis, MO, USA) and stained with ethidium bromide.

#### 4.2.2. PCR Amplification, Cloning and Sequencing

Internal Transcribed Spacer (ITS) Sequence Analysis of nuclear ribosomal DNA (nrDNA) was used to estimate the origin of putative hybrids. The complete Internal Transcribed Spacer assembly (ITS1-5.8S-ITS2) of the nuclear ribosomal DNA from *H. adriaticum, H. robertianum*, and its putative hybrids was amplified and sequenced, along with three plastid barcoding DNAs (*rbcl*, *matK* and *psbA-trnH* intergenic spacer). The complete Internal Transcribed Spacer assembly was amplified by the ITS5 and ITS4 universal primers [70] (Table 4). The PCR amplifications of all the DNA samples were always performed in duplicate. The amplification reaction was run in 20 µL containing approx. 30 ng of genomic DNA, 1 unit of DreamTaq polymerase (Thermo Fisher Scientific Baltics UAB, Vilnius, Lithuania), 1.5 mM MgCl_2_, 200 µM dNTPs, and 0.2 µM of both ITS primers. The amplification reactions were performed in a GeneAmp PCR System 2700 DNA thermal cycler (Applied Biosystems, Foster City, California, USA) using the following cycle profile: initial denaturation at 96 °C for 5 min followed by 35 cycles (95 °C for 45 s, 60 °C for 45 s and 72 °C for 1 min) and a final extension at 72 °C for 5 min. 

Amplification of plastid DNA was performed using the following thermal PCR conditions: initial denaturation at 95 °C for 5 min, followed by 35 cycles (95 °C for 30 s, 52–58 °C for 30 s, depending on the chosen primer pair, reported in Table 4 and an extension step at 72 °C for 45 s) and a final extension cycle at 72 °C for 5 min. The amplification products obtained from nuclear and plastid DNA were purified with a Qiaquick PCR Purification kit (Qiagen, Hilden, Germany) and sequenced at the Centro Interdipartimentale per le Biotecnologie Agrarie, Chimiche e Industriali (CIBIACI) of the University of Florence. The amplification product of the nuclear Internal Transcribed Spacer assembly (ITS1-5.8S-ITS2) of the hybrid *H. adriaticum* × *H. robertianum* was cloned into pTZ57R/T vector in a ligation mix (InsTAclone PCR Cloning Kit, Thermo Scientific^®^, Waltham, Massachusetts, USA) composed by the purified PCR fragment, 1X Ligation Buffer, T4 DNA ligase. The ligation mix was incubated at 22 °C overnight and later used to transform Top10 chemical competent bacteria. The bacterial colonies surviving the selective agent (ampicillin 100 mg L^−1^) were discriminated by the blue/white screening using X-gal dye system. The recombinant colonies were amplified with the ITS5 and ITS4 primers to identify those carrying the fragments of interest. Sixteen colonies, eight for each considered seedling, were selected and the plasmids were extracted and amplified with ITS primers. Finally, the amplification products were sequenced.

All nuclear and plastid sequences of the parental genotypes and the developed hybrids have been previously checked through data-mining sequences in GenBank ^®^ by using BLAST analysis, while those resulting from the present research have been submitted to GenBank (Table 5) The ITS and plastid barcoding sequences of the putative hybrids have been aligned with those of the parental plants using the Multalin software [71]. 

## 5. Conclusions

In the present study, anther preservation and in vitro germination of immature seeds were applied to obtain for the first time an interspecific hybrid between species belonging to the two subgenera *Barlia* and *Himantoglossum.* A protocol for in vitro germination and protocorms’ development of the hybrid was successfully achieved on both M551and BM-1 media under asymbiotic conditions, that is without the mycorrhization of the seeds by specific fungi, association which is essential during orchid seedling establishment and to its life cycle in natural habit. Our research showed that it is possible to obtain a large number of viable hybrid protocorms (over a thousand) from a single immature capsule. Seedling hardening, which is the crucial step after seed germination, proved to be successful as well. With an overall survival success just under 20%, production of plant material from *H. adriaticum* × *H. robertianum* through asymbiotic in vitro germination techniques offered opportunities for growing a sufficient number of seedlings for agronomic and phenotypic assessments of the new hybrid. 

Growth regulator applications and different substrate pH levels to induce in vitro multiplication and reduce degenerative phenomena, which were not utilized in this study, may prove to be advantageous for increasing number of subcultures and could be explored in further studies.

The hybrid interspecific origin of the protocorms was confirmed by the molecular analysis of the ITS1-5.8S-ITS2 regions of the nuclear ribosomal DNA and the plastid barcoding DNA sequences. The use of barcoding markers has been extensively used for the study of biodiversity [72] but is not a reliable tool for the investigation on the hybrid origin in phylogenetic studies of closely related clades because concerted evolution, via gene conversion or unequal crossing-over, can homogenize different parental genomes in a hybrid so that only one parental genome type may be seen in the hybrid [49,73,74]. Indeed, ITS barcoding could be a robust indicator of the parentage of taxa known from other evidence to be hybrids [49] such as supported by cytological data or, as in this case, by a recently made artificial pollination experiment. As far as we know, ITS barcoding has not been used so far to assess the parentage of the progeny of artificial hybridisation, although nrITS ribotype has been used successfully to elucidate phylogenetic relationships within the *Himantoglossum-steveniella* clade [75]. However, as soon as the hybrid will reach its first bloom, phenotypic analyses and agronomic tests will be carried out in order to evaluate its ornamental potential for commercial exploitation in the cut-flower/flowering pot/outdoor ornamental plant industry. Finally, the hybrid fertility will be evaluated along with the possibility to conduct the reciprocal cross (*H. robertianum* × *H. adriaticum*), which would implicate a long-term anther conservation (about 9 months) based on parental flowering periods.

## Figures and Tables

**Table 1:** Number of protocorms obtained from each seed capsule on the tested BM1 and M551 media. ^1^ Number of germinated protocorms are the average computed over 2 jars for each capsule × substrate combination. ^2^ Fungal contamination in pairs of jars.

Capsule N.	Sowing Date	Germinated Protocorms (N.) ^1^	
		BM1	M551
C1	21 June 2017	179	21
C2	27 June 2017	150	132
C3	27 June 2017	236	67
C4	21 June 2017	149	25
C5	26 June 2017	448	46
C6	26 June 2017	443	Contam. ^2^
C7	27 June 2017	170	157
C9	23 June 2017	311	69
C10	28 June 2017	391	928
C11	27 June 2017	145	247
C13	26 June 2017	133	74
C15	26 June 2017	211	94
C20	28 June 2017	160	878
C26	28 June 2017	398	1075
C59	28 June 2017	Contam. ^2^	1886
C62	28 June 2017	370	Contam. ^2^
Total		3894	5699

**Table 2:** Comparative informative sites of the ITS nuclear DNA sequences attained from the parents and from the colonies obtained after cloning the hybrids’ ITS amplicon. The exact bp position within the sequence of each nucleotide substitution has been indicated. The total length of the fragment is 686 bp, the nucleotide substitution sites are 49 with a percentage of 7.1%.

**Position**	**42**	**57**	**63**	**93**	**100**	**113**	**114**	**147**	**151**	**178**	**186**	**189**	**191**	**192**	**200**	**211**	**219**	**228**	**229**	**230**
*H. adriaticum*	T	T	C	G	T	T	T	C	T	T	A	T	C	A	T	C	T	C	T	T
colony 4	T	T	C	G	T	T	T	C	T	T	A	T	C	A	T	C	T	C	T	T
colony 7	T	T	C	G	T	T	T	C	T	T	A	T	C	A	T	C	T	C	T	T
colony 10	T	T	C	G	T	T	T	C	T	T	A	T	C	A	T	C	T	C	T	T
colony 11	T	T	C	G	T	T	T	C	T	T	A	T	C	A	T	C	T	C	T	T
*H. robertianum*	A	C	T	A	C	G	C	T	C	C	G	C	A	G	A	A	A	A	A	A
colony 5	A	C	T	A	C	G	C	T	C	C	G	C	A	G	A	A	A	A	A	A
colony 6	A	C	T	A	C	G	C	T	C	C	G	C	A	G	A	A	A	A	A	A
colony 13	A	C	T	A	C	G	C	T	C	C	G	C	A	G	A	A	A	A	A	A
colony 16	A	C	T	A	C	G	C	T	C	C	G	C	A	G	A	A	A	A	A	A
**Position**	**233**	**374**	**414**	**422**	**426**	**429**	**431**	**444**	**445**	**470**	**476**	**481**	**483**	**497**	**498**	**513**	**514**	**519**	**551**	**553**
*H. adriaticum*	C	G	A	T	A	T	A	T	T	G	T	T	C	G	C	T	T	C	A	T
colony 4	C	G	A	T	A	T	A	T	T	G	T	T	C	G	C	T	T	C	A	T
colony 7	C	G	A	T	A	T	A	T	T	G	T	T	C	G	C	T	T	C	A	T
colony 10	C	G	A	T	A	T	A	T	T	G	T	T	C	G	C	T	T	C	A	T
colony 11	C	G	A	T	A	T	A	T	T	G	T	T	C	G	C	T	T	C	A	T
*H. robertianum*	G	A	C	C	G	C	G	A	A	A	C	C	T	A	T	A	G	A	G	C
colony 5	G	A	C	C	G	C	G	A	A	A	C	C	T	A	T	A	G	A	G	C
colony 6	G	A	C	C	G	C	G	A	A	A	C	C	T	A	T	A	G	A	G	C
colony 13	G	A	C	C	G	C	G	A	A	A	C	C	T	A	T	A	G	A	G	C
colony 16	G	A	C	C	G	C	G	A	A	A	C	C	T	A	T	A	G	A	G	C
**Position**	**587**	**593**	**597**	**601**	**624**	**625**	**626**	**627**	**634**											
*H. adriaticum*	C	G	T	C	A	A	T	A	A											
colony 4	C	G	T	C	A	A	T	A	A											
colony 7	C	G	T	C	A	A	T	A	A											
colony 10	C	G	T	C	A	A	T	A	A											
colony 11	C	G	T	C	A	A	T	A	A											
*H. robertianum*	A	T	A	G	G	T	C	G	G											
colony 5	A	T	A	G	G	T	C	G	G											
colony 6	A	T	A	G	G	T	C	G	G											
colony 13	A	T	A	G	G	T	C	G	G											
colony 16	A	T	A	G	G	T	C	G	G											

**Table 3:** Comparative informative sites of the plastid DNA sequences (*matK*, *rbcL* and *psbA-trnH*) obtained from direct sequencing of the parents and the hybrids as template. The exact bp position within the sequence of each nucleotide substitution, deletion and insertion has been indicated. The total length of the fragment in bp and the number of nucleotide mutation sites have also been reported for each plastid barcode sequence.

	*matK* 11/830 bp											*rbcL* 2/610 bp						
**Position**	**18**	**78**	**208**	**300**	**350**	**616**	**678**	**696**	**718**	**773**	**812**		**251**	**259**				
*H. robertianum*	A	A	T	C	G	G	C	G	C	G	A		G	A				
*H. adriaticum*	G	G	C	T	A	T	T	A	T	A	G		A	T				
Hybrid 6	G	G	C	T	A	T	T	A	T	A	G		A	T				
Hybrid 11	G	G	C	T	A	T	T	A	T	A	G		A	T				
	***psbA-trnH*** **36/813bp**																	
**Position**	**58**	**59**	**60**	**61**	**62**	**63**	**91**	**92**	**93**	**94**	**95**	**96**	**141**	**142**	**143**	**144**	**145**	**146**
*H. robertianum*	-	-	-	-	-	-	-	-	-	-	-	-	T	T	T	C	T	C
*H. adriaticum*	T	C	C	C	C	A	G	G	G	A	T	G	-	-	-	-	-	-
Hybrid 6	T	C	C	C	C	A	G	G	G	A	T	G	-	-	-	-	-	-
Hybrid 11	T	C	C	C	C	A	G	G	G	A	T	G	-	-	-	-	-	-
	***psbA-trnH*** **36/813 bp**																	
**Position**	**147**	**148**	**149**	**242**	**243**	**244**	**245**	**246**	**247**	**248**	**249**	**250**	**172**	**196**	**260**	**274**	**336**	**720**
*H. robertianum*	C	G	A	A	G	A	A	A	A	G	T	C	G	G	C	G	T	A
*H. adriaticum*	-	-	-	-	-	-	-	-	-	-	-	-	T	T	G	T	A	C
Hybrid 6	-	-	-	-	-	-	-	-	-	-	-	-	T	T	G	T	A	C
Hybrid 11	-	-	-	-	-	-	-	-	-	-	-	-	T	T	G	T	A	C

**Table 4:** List and sequence of the primers used for the amplification of the barcoding markers with the respective annealing temperature.

Locus	Primer Name	Sequence (5’ → 3’)	Annealing T
***rbcL***	F1	ATGTCACCACAAACAGAGACTAAAGC	58 °C
	R634	GAAACGGTCTCTCCAACGCAT	
***matK***	3F	CGTACAGTACTTTTGTGTTTACGAG	52 °C
	1R	ACCCAGTCCATCTGGAAATCTTGGTTC	
***psbA-trnH***	Fwd	CGCGCATGGTGGATTCACAATCC	55 °C
	Rev	GTTATGCATGAACGTAATGCTC	
**ITS**	ITS5 F	GGAAGTAAAAGTCGTAACAAGG	50 °C
	ITS4 R	TCCTCCGCTTATTGATATGC	

**Table 5:** The nucleotide sequence data reported (ITS, *matK*, *rbcl* and *psbA-trnH* spacer of the two *Himantoglossum* species) are available in the GenBank database under the accession numbers listed in the table. Asterisks indicate the accession number of the sequences not available in GenBank and deposited for the present publication.

	ITS	*matK*	*rbcl*	*psbA-trnH* Spacer
*Himantoglossum adriaticum*	FR750401	MW367975 *	MW367974 *	MW316730 *
*Himantoglossum robertianum*	KJ596109	AY368382	AY368337	MW316729 *

## Data Availability

Data is contained within the article or supplementary material.

## Supplementary Materials

The following are available online at https://www.mdpi.com/2223-7747/10/1/107/s1, Figure S1: Agarose gel electrophoresis of the ITS amplification of the samples: 1 = *H. adriaticum*; 2 = *H. robertianum*; 3 and 4 = putative hybrid lines; 5 = molecular marker; 6 = no template control. Figure S2: *H. robertianum* pollinaria after dehydration with silica gel and storage for 70 days in the dark at 4 °C. Figure S3: Detail of the stigmatic cavity of a manually emasculated *H. adriaticum* flower pollinated by rubbing an entire pollinarium of *H. robertianum* (inside the circle).
