# Supplementary figures and images for: Himantoglossum adriaticum H. Baumann × Himantoglossum robertianum (Loisel.) P. Delforge: A New Interspecific Hybrid Assessed by Barcoding Analysis
Source: Plants (Basel). 2021 Jan 6;10(1):107. doi: 10.3390/plants10010107 (PMC7825628; doi:10.3390/plants10010107)

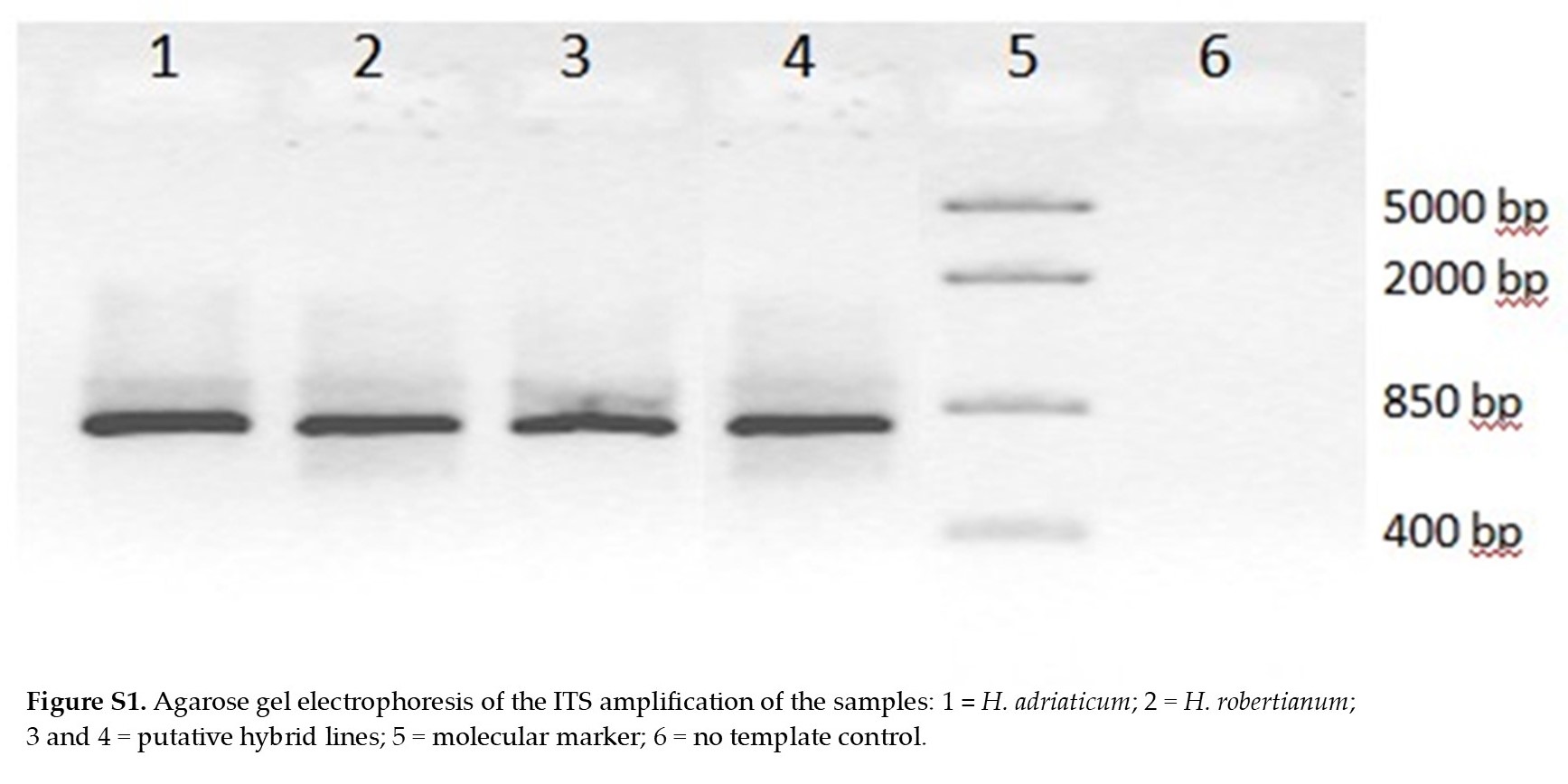

Supplement: Supplementary file 1 [file plants-10-00107-s001.zip › SUPPLEMENTARY FILES/Figure S1.jpg]

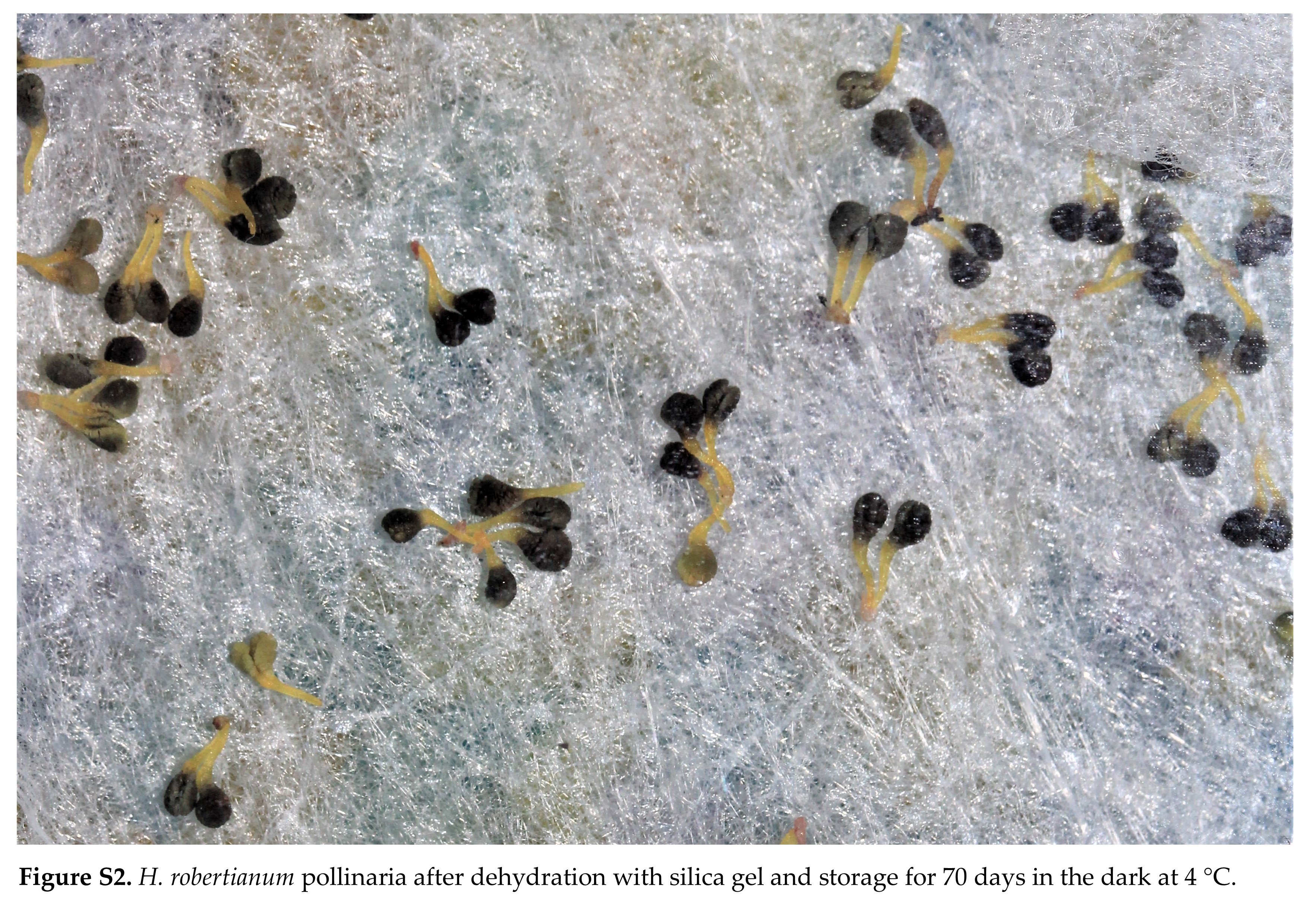

Supplement: Supplementary file 1 [file plants-10-00107-s001.zip › SUPPLEMENTARY FILES/Figure S2.jpg]

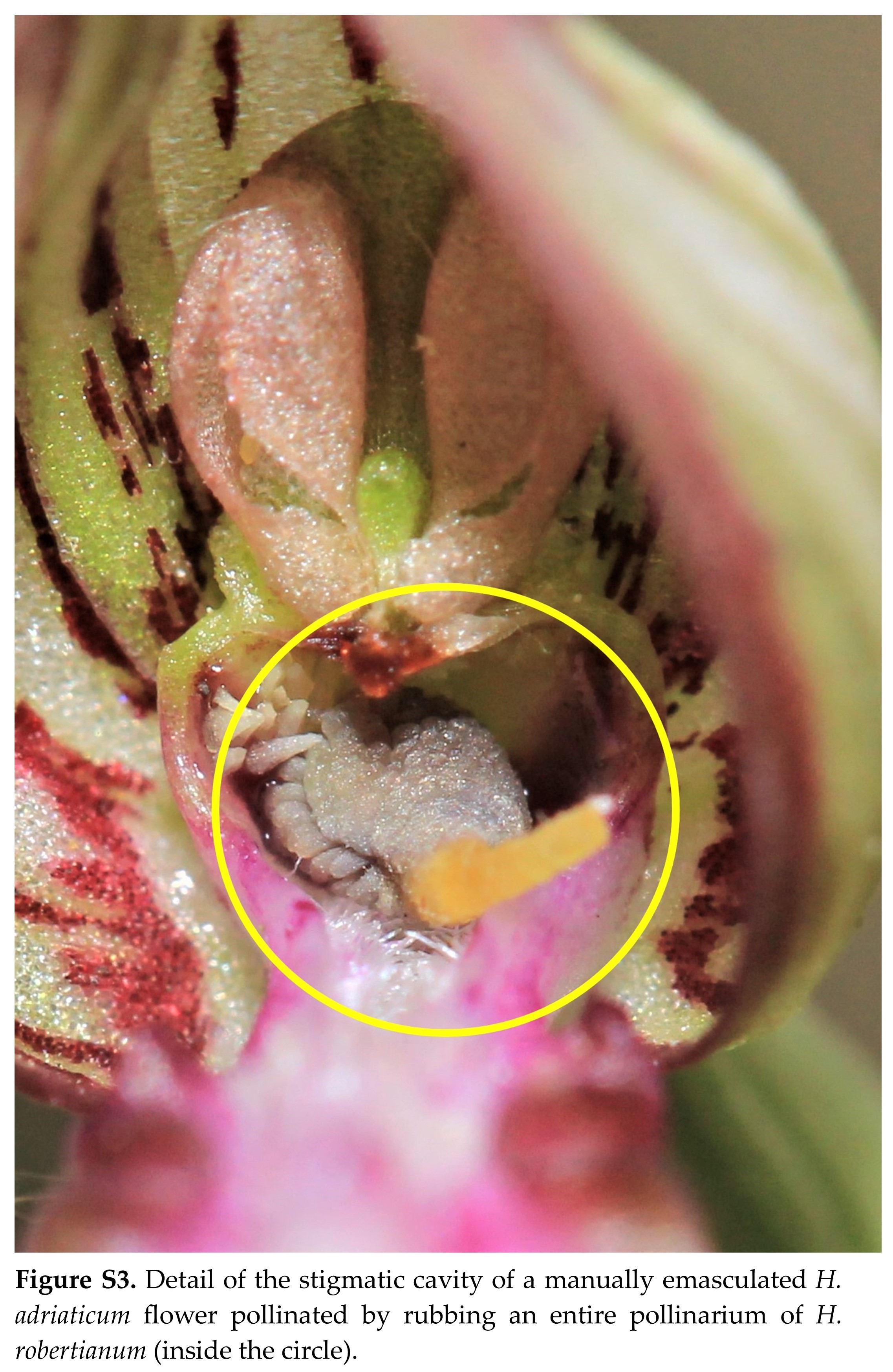

Supplement: Supplementary file 1 [file plants-10-00107-s001.zip › SUPPLEMENTARY FILES/Figure S3.jpg]
